# Effects of ALA-PDT on the Healing of Mouse Skin Wounds Infected With *Pseudomonas aeruginosa* and Its Related Mechanisms

**DOI:** 10.3389/fcell.2020.585132

**Published:** 2020-12-04

**Authors:** Tao Yang, Yang Tan, Wentao Zhang, Weijiang Yang, Jiefu Luo, Ling Chen, Hong Liu, Guihong Yang, Xia Lei

**Affiliations:** Department of Dermatology, Daping Hospital, The Army Medical University, Chongqing, China

**Keywords:** photodynamic therapy, wound healing, *Pseudomonas aeruginosa*, macrophagocyte, inflammatory factor

## Abstract

Photodynamic therapy (PDT) is a promising new method to eliminate microbial infection and promote wound healing. Its effectiveness has been confirmed by some studies; however, the mechanisms of PDT in wound healing remain obscure. We used mouse skin wounds infected with *Pseudomonas aeruginosa* as a research object to explore the therapeutic effects and mechanisms of 5-aminolevulinic acid photodynamic therapy (ALA-PDT). ALA-PDT treatment significantly reduced the load of *P. aeruginosa* in the wound and surrounding tissues and promoted the healing of skin wounds in mice. Hematoxylin-eosin (HE) and Sirius red staining showed that ALA-PDT promoted granulation tissue formation, angiogenesis, and collagen regeneration and remodeling. After ALA-PDT treatment, the expression of inflammatory factors (TNF-α and IL-1β) first increased and then decreased, while the secretion of growth factors (TGF-β-1 and VEGF) increased gradually after treatment. Furthermore, ALA-PDT affected the polarization state of macrophages, activating and promoting macrophages from an M1 to an M2 phenotype. In conclusion, ALA-PDT can not only kill bacteria but also promote wound healing by regulating inflammatory factors, collagen remodeling and macrophages. This study further clarifies the mechanism of PDT in the healing of infectious skin wounds and provides further experimental evidence for its clinical treatment of skin wounds infected by *P. aeruginosa*.

## Introduction

Skin wounds are a common refractory disease in dermatology and occur in conjunction with microbial infection. The treatment of skin wounds usually includes systemic antibiotics, localized treatment with conventional clinical dressings, irrigation, negative pressure drainage, and surgical operation. However, these treatments used to address skin wounds are always time-consuming and expensive. Currently, there have been increasing reports of *Pseudomonas aeruginosa* infections resulting from skin wounds. *P. aeruginosa* is both highly resistant and naturally unsusceptible to many antibiotics and tends to form biofilms on infected surfaces.

Photodynamic therapy (PDT) has been shown to be a very successful therapy in clinical practice and is widely used in the treatment of certain skin diseases, such as acne, viral warts, and skin cancers (St. Denis et al., 2014). Currently, PDT is a promising new method used to eliminate microbial infections because it can produce photochemical reactions using photosensitizers and light, producing singlet oxygen, which is toxic and inactivates target microorganisms. Some studies have confirmed that PDT is very effective against bacterial and fungal infections (Jori et al., 2006; Wardlaw et al., 2012; Sobotta et al., 2019). Additionally, PDT is considered an important innovative alternative treatment for the healing of skin wounds (Nesi-Reis et al., 2018) that can reduce treatment time and promote healing. However, the mechanisms of PDT in wound healing remain obscure.

In this study, we used mouse skin wounds infected with *P. aeruginosa* as a research model of infected skin wounds. The results showed that aminolevulinic acid photodynamic therapy (ALA-PDT) can not only kill bacteria but also promote wound healing by regulating inflammatory factors, collagen remodeling and macrophages.

## Materials and Methods

### Establishment of Infected Skin Wounds on Model Animals

Sixty healthy Kunming mice weighing 30–40 g and aged 4–5 weeks were provided by the Animal Center of Daping Hospital (Army Military Medical University). One week before the experiment, the mice were house in the experimental environment at a room temperature of 20–25°C.

*Pseudomonas aeruginosa* (ATCC 27853) was stored at −80°C, and the cultures were maintained on Luria Bertani (LB) plates at 37°C with agitation (200 rpm). The bacterial suspensions were diluted to an optical density of 0.5 (approximately 2 × 10^8^ CFU/mL bacteria) in normal saline, as shown by measurement with a turbidimetric instrument.

A model of acute bacterial infection was established after full-thickness skin excision. The mice were anesthetized by intraperitoneal injection of 10% chloral hydrate at a dose of 300 mg/kg. An area of the back (3 cm × 4 cm) was shaved. After disinfection with iodophor, two round symmetrical marks with a diameter of 10 mm were made in the skin along both sides of the spine, and each circle had an area of 0.79 cm^2^. Full-thickness skin defects were made along the indentation with aseptic curved surgical scissors without damaging the fascial layer or muscle. A 100-μL suspension of *P. aeruginosa* (1 × 10^8^ strains) was dripped into each wound. The wound was fixed with gauze, and the mice were divided into four groups housed in individual cages. The duration from inoculation to successful modeling is 24 h. After successful modeling, a green purulent secretion was found on the surface of the wounds on the mice, the temperature of the skin around the wound was increased, the behavior of the mice was depressed, the mice showed a poor appetite, and some mice had loose stools. After 7 days of treatment, these symptoms of mice in each group were significantly recovered, and there was no significant difference between the groups.

All the animal care and experimental protocols were reviewed and approved by the Laboratory Animal Welfare and Ethics Committee of Third Military Medical University. All the experiments were performed in accordance with the relevant guidelines for the care and use of laboratory animals.

### ALA-PDT Treatment

5-Aminolevulinic acid (ALA) (Fudan Zhangjiang Company, Shanghai, China) was used as the photosensitizer with an incubation of 30 min. A stock solution was prepared by dissolving ALA powder in normal saline to a final concentration of 1.408 mol/L. A 630-nm LED (Omnibus, United Kingdom) light source was used to activate the ALA, and the output power density was 54 J/cm^2^ (the laser power used was 90 MW/cm^2^, and the treatment time was 10 min.). There were four experimental groups: the control group (without ALA or light irradiation), the red light group (light dose of 54 J/cm^2^ without ALA), the ALA group (1.408 mol/L ALA with no light irradiation), and the ALA-PDT group (1.408 mol/L ALA with a light dose of 54 J/cm^2^).

The mice were anesthetized and fixed for 24 h after infection. The mice in each group were bandaged, fed regularly and observed until the end of the corresponding time point of the experiment.

### Counting of Bacteria on Wounds

The number of bacteria on the wound surface before treatment (day 0) and on the 1st, 3rd, 7th, and 14th days after treatment was measured. At each time point, the tissue at the wound edge for each group was cut, weighed, added to normal saline at a concentration of 0.01 g/ml and homogenized in a sterile homogenizer. For detection, the sample was diluted sixfold in normal saline, and 10 μl of homogenate from each dilution was evenly spread on two ordinary broth agar plates. The colony morphology was observed, and the colonies were counted.

### Wound Healing Assay

A digital camera was used to take pictures before treatment (day 0) and on the 1st, 3rd, 7th, and 14th days after treatment, and the wound area and wound healing rate were calculated with standard grid paper as follows: wound healing rate (%) = (wound area before treatment−wound area after treatment)/wound area before treatment.

### HE Staining

Before treatment (day 0) and on the 1st, 3rd, 7th, and 14th days after treatment, granulation tissue under the new epithelium was taken from the infected wounds of the mice under sterile conditions, immediately cut into 0.5 cm × 0.5 cm tissue blocks, and fixed in a 4% polycarboxylic acid solution. After conventional dehydration and clearing, the blocks were embedded in paraffin. The slices were 3–5-μm thick. A general optical microscope was used to observe inflammation, fibroblasts, the number of capillaries, and the degree of wound contraction and re-epithelialization after hematoxylin-eosin (HE) staining.

### Sirius Red Polarized Light Assay

Before treatment (day 0) and on the 1st, 3rd, 7th, and 14th days after treatment, granulation tissue under the new epithelium was taken from the infected wounds of the mice under sterile conditions, immediately cut into 0.5 cm × 0.5 cm tissue blocks, and fixed in a 4% polycarboxylic acid solution. After conventional dehydration and clearing, the blocks were embedded in paraffin. The slices were 3–5-μm thick. Then, polarized light microscopy was used to observe collagen remodeling after picric acid/Sirius red staining. Under a polarized light microscope, type I collagen fibers showed strong birefringence and were red or orange-yellow. Type III collagen fibers showed weak birefringence and were green and thin.

### Western Blot Analysis

On the 3rd, 7th, and 14th days after treatment, granulation tissue under the new epithelium was taken from the infected wounds of the mice under sterile conditions. Nuclear and cytoplasmic protein lysates were prepared. The protein concentrations were measured with a BCA Protein Assay Kit (Beyotime, Nanjing, China). Equal amounts of protein (30 μg) were loaded into each lane of a 10% sodium dodecyl sulfate polyacrylamide gel electrophoresis (SDS-PAGE) gel and separated. After the proteins had been transferred onto polyvinylidene difluoride membranes, the membranes were blocked and incubated with primary antibodies at 4°C overnight. The following primary antibodies were used: anti-TNF-α, anti-IL-1β, anti-TGFβ-1, and anti-VEGF antibodies from Abcam (United States) and anti-β-actin antibody from Bioworld (United States). After incubation with secondary antibody (goat anti-rabbit), the membranes were treated with an enhanced chemiluminescence reagent mixture (Thermo Fisher Scientific, United States) for 5 min and imaged on a Vilber Lourmat Fusion FX5 system (China).

### Immunofluorescence Staining

M1 and M2 macrophage polarization was observed by immunofluorescence staining. The marker of M1 and M2 macrophages are iNOS and CD163, respectively. The primary antibodies anti-iNOS (rabbit polyclonal antibody, 1:100) and anti-CD163 (goat polyclonal antibody, 1:100) from Servicebio (China) and the fluorescent second antibodies goat anti-rabbit (1:300) and rabbit anti-goat (1:300) from Servicebio (China) and DAPI from Abcam (United States) were used. After immunofluorescence staining, the paraffin sections were observed under a fluorescence microscope, and images were collected (DAPI emits blue light at 330–380 and 420 nm; FITC emits green light at 465–495 and 515–555 nm; Cy3 emits red light at 510–560 and 590 nm.). Eight visual fields were randomly selected from each slide, and three separate repeated experiments were conducted. All cells in the visual field were included in the statistical analysis.

### Statistical Analyses

All experiments were independently performed with three replicates. The results are presented as the mean ± SD. Statistical differences were analyzed by one-way ANOVA with SPSS Statistics 20.0 (IBM, United States). *P*-values less than 0.05 were considered to indicate statistically significant differences.

## Results

### Effects of ALA-PDT on *P. aeruginosa* Growth on the Wounds

**FIGURE 1:**
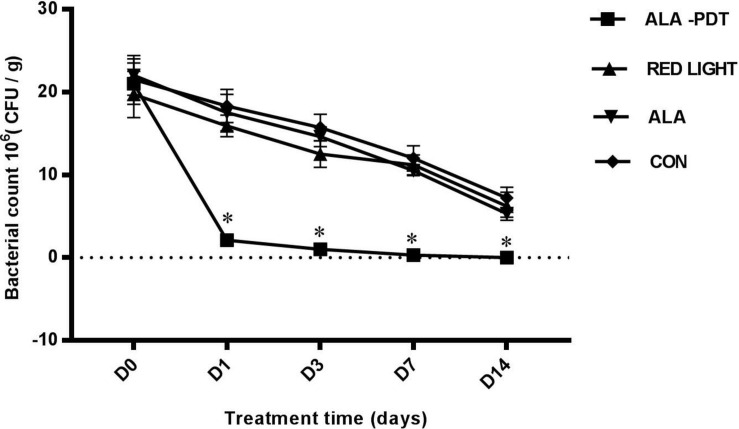
Bacterial counting on wounds. The amounts of *P. aeruginosa* were counted on wounds before treatment (D0) and on the 1st (D1), 3rd (D3), 7th (D7), 14th (D14) day after different treatments. ^∗^*P* < 0.05 versus the other groups.

To investigate the antibacterial effect of ALA-PDT, we detected the amount of *P. aeruginosa* on the wounds before treatment and on the 1st, 3rd, 7th, and 14th days after different treatments. The results are shown in Table 1. No significant differences in bacterial growth were observed in the control, light and ALA groups. The ALA-PDT group showed a significant antibacterial effect and significantly less bacteria relative to that in the control group (*P* < 0.05), indicating that ALA-PDT inhibited *P. aeruginosa* growth (Figure 1).

**TABLE 1:** Results of *P. aeruginosa* growth on the wounds (units: 10^6^CFU/g).

	Control	ALA	Red light	ALA-PDT
Day 0	21.5 ± 2.5	22.0 ± 2.4	19.7 ± 2.8	21.0 ± 2.5
Day 1	18.3 ± 2.0	17.5 ± 2.2	15.9 ± 1.3	2.1 ± 0.1*
Day 3	15.7 ± 1.6	14.6 ± 1.2	12.5 ± 1.6	1.0 ± 0.1*
Day 7	12.0 ± 1.5	10.5 ± 0.6	11.2 ± 1.2	0.3 ± 0.1*
Day 14	7.2 ± 1.3	5.3 ± 0.4	6.2 ± 1.7	0.0 ± 0.0*

*P < 0.05 versus the other groups.

### Effects of ALA-PDT on the Wounds Healing Rate

**FIGURE 2:**
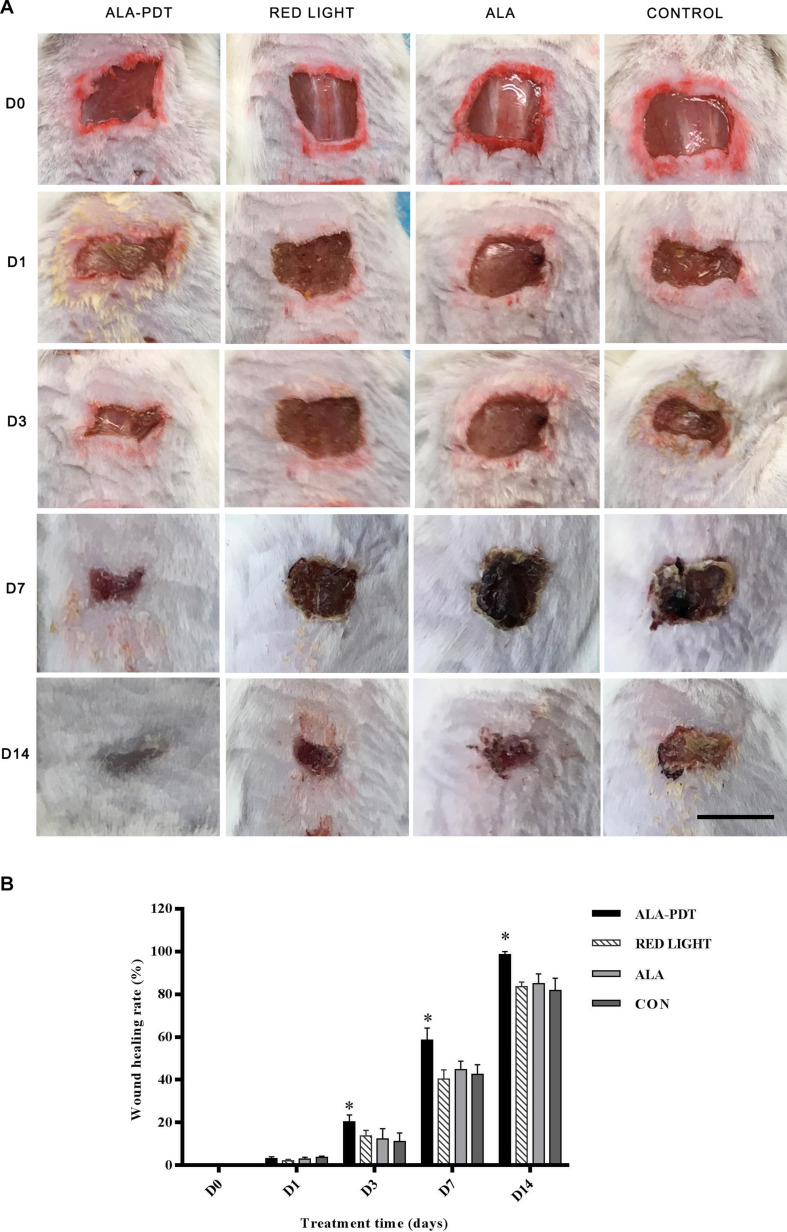
Effects of ALA-PDT for the healing rate on wounds. **(A)** The wound pictures of healing process. Scale bar indicate 1 cm. **(B)** The healing rate was counted before treatment (D0) and on the 1st (D1), 3rd (D3), 7th (D7), 14th (D14) day after different treatments. ^∗^*P* < 0.05 versus the other groups.

To investigate the effect of ALA-PDT on wound healing, we observed the wound healing rates on the 1st, 3rd, 7th, and 14th days after different treatments. The results are shown in Table 1. No significant differences in wound healing rate were observed in the control, light and ALA groups. Furthermore, there were no significant differences in wound healing among the groups on 1st day after treatment. The ALA-PDT group showed significantly increased wound healing relative to that in the control group (*P* < 0.05) on the 3rd, 7th, and 14th days (Figure 2).

### Observation of the Effects of ALA-PDT on Wounds by HE Staining

**FIGURE 3:**
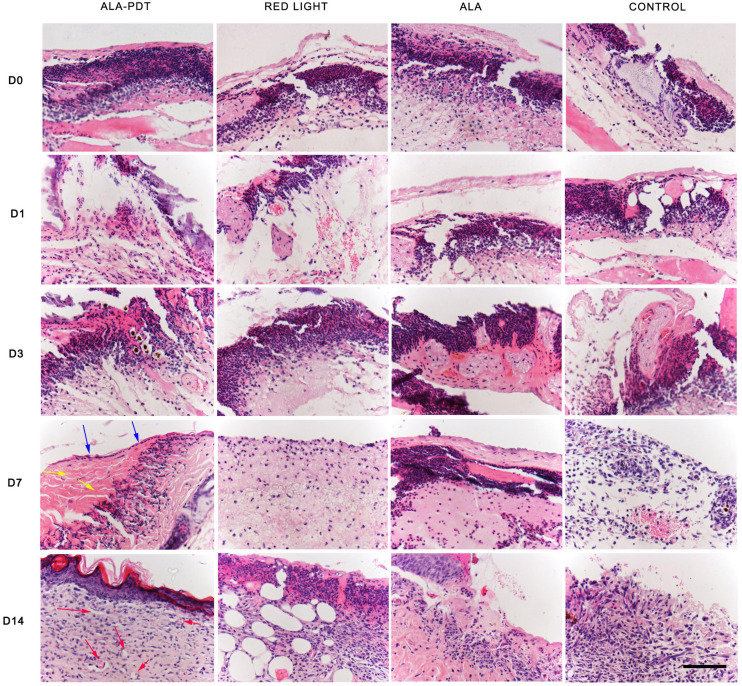
HE staining assay. The HE staining results of each group before treatment (D0) and on the 1st (D1), 3rd (D3), 7th (D7), 14th (D14) day after different treatments. The blue arrows indicate the structure of the new epithelium. The yellow arrows indicate relatively thickened and increased collagen fibers. The red arrows indicate new capillaries. Scale bar indicate 100 μm.

The HE staining results on the 1st and 3rd days after treatment did not significantly differ among the groups. On the 7th day after treatment, obvious ulcer formation was found in the control group, light group and ALA group, there was no epithelioid hyperplasia, and a large number of inflammatory cells had accumulated in the periwound tissues. In the ALA-PDT group, more granulation tissue and neovascularization were observed, and only the superficial dermis contained inflammatory cells; on the 14th day after treatment, some skin defects remained in the control group, light group and ALA group, which contained blisters, blood, scabs, remaining inflammatory cells and granulation tissue formation. In the ALA-PDT group, epithelial cells, and fibrous tissue proliferation were clearly observed, and only some inflammatory cells had infiltrated the tissue around blood vessels (Figure 3).

### Sirius Red Polarized Light Observation of the Effects of ALA-PDT on Wounds

**FIGURE 4:**
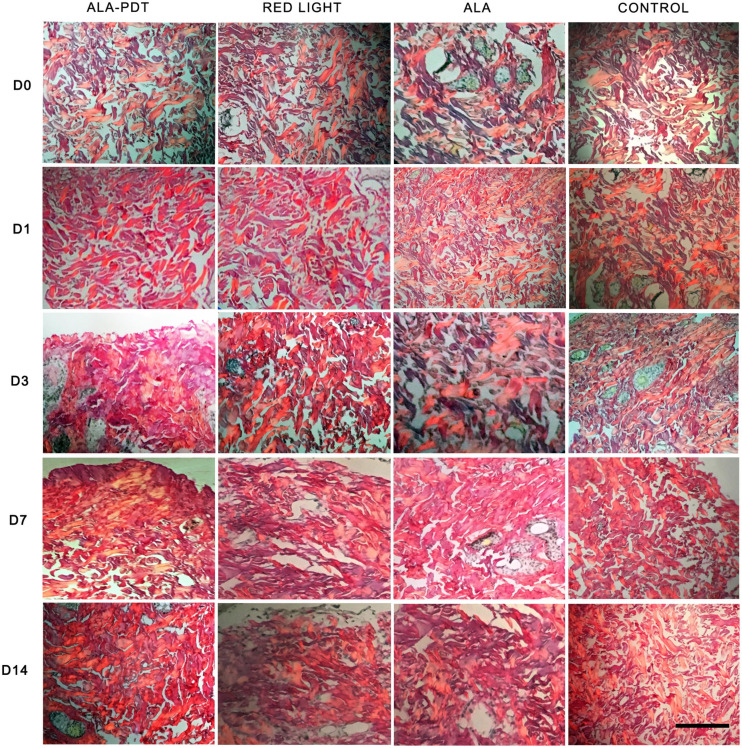
Sirius red polarized light method assay. The picric acid Sirius red staining results of each group before treatment (D0) and on the 1st (D1), 3rd (D3), 7th (D7), 14th (D14) day after different treatments. Scale bar indicate 50 μm.

Before treatment (day 0) and on the 1st day after treatment, collagen in each group was short and orderly arranged. On the 3rd day after treatment, the collagen fibers distributed in groups were thickened and increased in varying degrees in each group. Compared with control group, ALA group and red light group, the collagen distribution in ALA-PDT group was more dense. On the 7th day after treatment, compared with that in the control group, ALA group and light group, the collagen in the ALA-PDT group was thicker and denser, and unlike the other groups, the ALA-PDT exhibited collagen distributed in bundles. On the 14th day after treatment, compared with that in the control group, ALA group, and light group, the morphology of the collagen in the ALA-PDT group was coarser, and the collagen was more uniform (Figure 4).

### Western Blot Analysis of TNF-α, IL-1β, TGFβ-1, and VEGF Levels in Wounds

**FIGURE 5:**
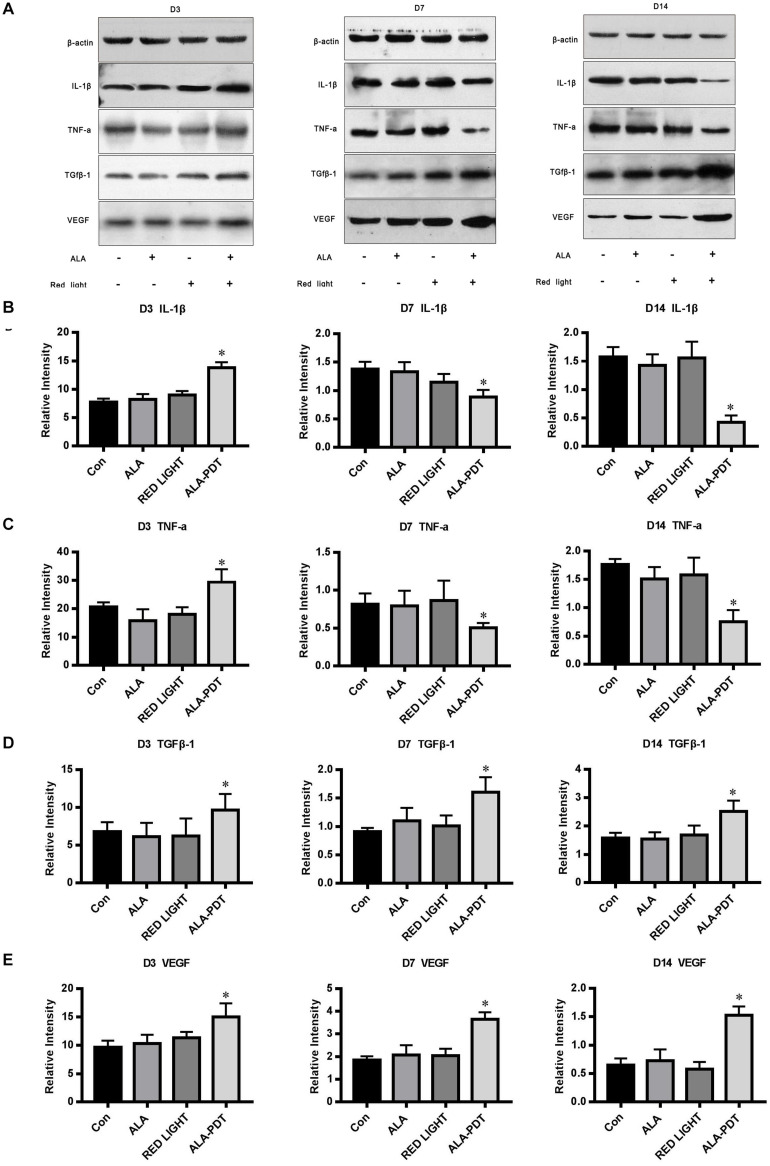
Effects of ALA-PDT on TNF-α, IL-1β, TGFβ-1, and VEGF. **(A)** Western blot assay of TNF-α, IL-1β, TGFβ-1, and VEGF proteins on the 3rd (D3), 7th (D7), 14th (D14) day after different treatments in wounds. **(B)** Western blot analysis of IL-1β. ^∗^*P* < 0.05 versus the other groups. **(C)** Western blot analysis of TNF-α. ^∗^*P* < 0.05 versus the other groups. **(D)** Western blot analysis of TGFβ-1. ^∗^*P* < 0.05 versus the other groups. **(E)** Western blot analysis of VEGF. ^∗^*P* < 0.05 versus the other groups.

To determine the mechanism by which ALA-PDT promotes wound healing, we further investigated the levels of TNF-α, IL-1β, TGFβ-1, and VEGF in wounds. Compared to the control group, red light group, and ALA group, the levels of TNF-α and IL-1β in the ALA-PDT group were higher on the 3rd day after treatment (*P* < 0.05) and lower on the 7th and 14th days after treatment (*P* < 0.05). Compared to the control group, red light group, and ALA group, the levels of TGFβ-1 and VEGF in the ALA-PDT group were higher than those in the control group on the 3rd, 7th, and 14th days after treatment (*P* < 0.05) (Figure 5).

### Effects of ALA-PDT on M1 and M2 Macrophages in Wounds

To gain further insight into the pro-healing effect of ALA-PDT on wounds, we determined the numbers of M1 and M2 macrophages in wounds. Cells stained red were considered M1 macrophages, and cells stained green were considered M2 macrophages.

**FIGURE 6:**
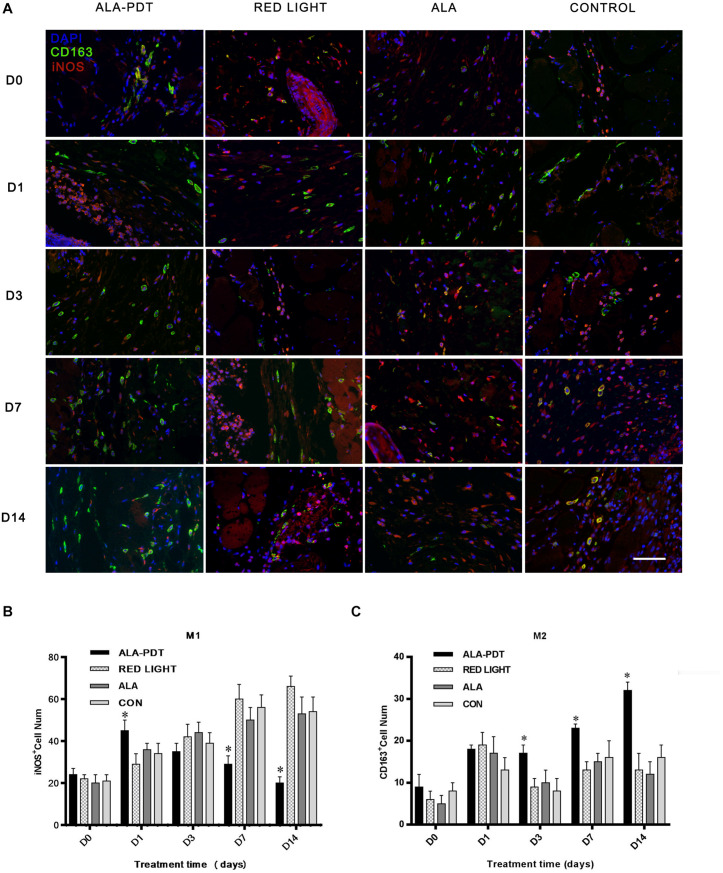
Effects of ALA-PDT on M1 and M2 macrophages in wounds. **(A)** Immunofluorescence staining of M1 and M2 macrophages before treatment (D0) and on the 1st (D1), 3rd (D3), 7th (D7), 14th (D14) day after treatment in wounds. The primary antibodies anti-iNOS (rabbit polyclonal antibody, 1:100) and anti-CD163 (goat polyclonal antibody, 1:100) from Servicebio (China) and the fluorescent second antibodies goat anti-rabbit (1:300) and rabbit anti-goat (1:300) from Servicebio (China) and DAPI from Abcam (United States) were used. The marker of M1 and M2 macrophages are iNOS and CD163, respectively. Cells stained red were considered M1 macrophages, and cells stained green were considered M2 macrophages. **(B,C)** Immunofluorescence staining analysis of M1 and M2 macrophages. ^∗^*P* < 0.05 versus the control groups. Scale bar indicate 20 μm.

Immunofluorescence staining showed that the number of M1 macrophages in ALA-PDT group was significantly increased compared with the control group on the 1st day, while on the 7th and 14th day, the number of M1 macrophages in ALA-PDT group was significantly lower than that in control group. On 1st, 3rd, 7th, and 14th day, the number of M2 macrophages in ALA-PDT group was significantly higher than that in control group (Figure 6).

## Discussion

Photodynamic therapy is a promising new method used to eliminate microbial infection and an important innovative alternative treatment for healing skin wounds. The treatment of infected skin wounds, including skin ulcers, skin abscesses, and skin sinus tracts, has advanced, but the mechanism by which PDT induces healing remains unclear.

Skin-infected wounds are a common refractory disease in dermatology that seriously affects patient quality of life and is costly. Infections with *P. aeruginosa*, a Gram-negative bacterium, are difficult to treat. *P. aeruginosa* is a common pathogen in serious skin wounds and infections of the urinary and respiratory systems. Tan et al. (2018) reported that ALA-PDT can kill planktonic and viable biofilm-associated *P. aeruginosa* cells, destroy biofilm structures, reduce virulence factor secretion, and affect QS system gene expression. Additionally, the incidence of *P. aeruginosa* infection resulting from skin wounds is increasing. Therefore, in this study, we used skin wounds in mice infected with *P. aeruginosa* as a research model of infected skin wounds.

The principle of PDT is in the administration of a photosensitiser (PS), followed by irradiation of the lesion with visible light in the presence of molecular oxygen, which generates a variety of ROS. The mechanisms by which ROS induce direct cell death through different pathways, including apoptosis, necrosis and/or autophagy, depend on the type of cell treated, dose of light supplied, concentration of PS used and intracellular location of the PS (Agostinis et al., 2011). PDT has been widely used in dermatology to treat certain skin cancers, viral warts, vascular diseases, and acne. With further in-depth research, an increasing number of studies have found that PDT is very effective against microbial infection and can promote the healing of infected skin wounds, especially skin wounds or skin ulcers infected by resistant or rare bacteria. Lei et al. (2015) reported that when half of 26 lower limb ulcers infected with *P. aeruginosa* were treated by PDT, PDT had significant antibacterial and pro-healing effects on the infected ulcers. Brown (2012) reported the potential of PDT to accelerate wound healing and prevent clinical infection. Morley et al. (2013) reported a randomized controlled trial of PDT for the treatment of chronic ulcers, the results of which suggested that PDT significantly reduced the wound bacterial load and clearly tended to promote healing in the PDT group.

The basic physiological process of cutaneous healing is dynamic and complex, involving a diverse set of molecular, cellular, and biochemical events that culminate in the reconstruction of damaged tissue. Wound healing involves a variety of cells, including inflammatory cells, fibroblasts, keratinocytes, and endothelial cells, which produce numerous inflammatory cytokines such as IL-1β and TNF-α (Werner and Grose, 2003). The ability of PDT to effectively eradicate microorganisms contributes significantly to the wound healing process. In this study, the load of *P. aeruginosa* was rapidly reduced after PDT treatment. PDT can also directly affect the process of wound healing. Inflammation is one of the steps involved in the mechanism of PDT in wound healing. Inflammation is divided into acute inflammation and chronic inflammation. Acute inflammation plays a beneficial role against infection and lesions (Kundu and Surh, 2012), contributing to wound healing, while chronic inflammation inhibits the healing process. The inflammatory process is a crucial part of healing since some inflammatory mediators at low-concentrations are related to cell proliferation and migration and also to the host defense against pathogens (Li et al., 2007; Liu et al., 2013). It has been reported that high TNF-α level inhibit angiogenesis (Fajardo et al., 1992) and wound repair is promoted due to inhibition of TNF-α (Mori et al., 2002). PDT can intervene by stimulating acute inflammation, contributing to changes in the physiological processes in chronic wounds, regardless of whether they are infected, promoting healing (Devirgiliis et al., 2011; Cappugi et al., 2014; Lei et al., 2015). Recent studies indicate that PDT induces a localized acute inflammatory response, leading to activation of the immune system (Anzengruber et al., 2015). Neutrophils are the first immune cells to arrive at a lesion, which is facilitated by the generation of TNF-α (Gollnick and Brackett, 2010; Cappugi et al., 2014). Other studies have also shown that PDT has a significant impact on neutrophil activation (Brackett and Gollnick, 2011; Gollnick, 2012), which can contribute to the increase in pro-inflammatory cytokines after PDT (Domínguez et al., 2010; Mroz and Hamblin, 2011). Chronic and persistent inflammation is a hallmark of most chronic wounds, but the relationships between chronic inflammation and PDT have scarcely been reported in the literature. The results of this study indicated increased acute inflammation in the early stage of wound healing and decreased chronic inflammation in the middle and later stages of wound healing in the PDT treatment group compared with the other groups. Through a comparative study of HE staining in the wound tissues in each group at different time points after treatment, it was found that the acute inflammatory reaction was more pronounced in the PDT treatment group than the other groups, accompanied by tissue edema in the early stage of treatment (1–3 days), while the chronic inflammatory reaction was significantly decreased in the PDT treatment group compared with the other groups in the middle and later stages of the healing process (7–14 days). The number of new capillaries increased sharply, and the collagen fibers were also bulkier and more obvious in the PDT treatment group compared to the other groups in the middle and later stages of the healing process. In this study, the expression of TNF-α and IL-1β in first increased and then decreased in the PDT treatment group; that is, the expression of TNF-α and IL-1β was higher in the acute inflammatory stage, while their expression was lower in the chronic stage in the PDT treatment group compared to the other groups. Therefore, PDT can promote wound healing in both the acute and chronic inflammatory stages.

Lesions release mediators (leukocytes and platelet migration) that start the repair process by first stimulating the inflammatory process. In sequence, the proliferative stage, in which re-epithelization, angiogenesis and an increase in fibroblast numbers occur, is first observed. The early onset of wound re-epithelialization after PDT, with the presence of young fibroblasts, fibrin, and granulation tissue, has been described by studies in animal models (Garcia et al., 2010; Sperandio et al., 2010). Garcia et al. (2010) described the positive effects of PDT on collagen deposition, proliferation, and angiogenesis in 3rd degree burns. In this study, the number of new capillaries increased rapidly, and the collagen fibers were thicker, denser, and distributed in bundles after PDT treatment. These results suggested that ALA-PDT can stimulate collagen proliferation and angiogenesis, promoting wound healing.

In the process of proper wound remodeling, balance between the synthesis and degradation of extracellular matrix is required, and it is evident that PDT modulates the production of TGF-β (Faffar et al., 2011). Studies of excisional wounds in human skin revealed an increase in TGF-β1 and β3, promoting the ordered deposition in collagen fibers (Mills et al., 2014). In this study, the expression levels of TGF β-1 and VEGF in the PDT treatment group and other groups tended to be increased in the healing process, while their expression levels were significantly higher in the PDT treatment groups than in the other groups at the same time. Compared with VEGF expression, TGF β-1 expression was most obviously increased in the 20% ALA-PDT group, suggesting that PDT regulates and activates cell growth factors, which would more deeply affect the wound tissue repair process.

The roles of inflammatory cytokines and the inflammatory response in the mechanism of bacterial infection after wound formation cannot be ignored. These inflammatory factors are partly produced by macrophages, which can be activated by monocytes. Macrophage activation is a continuous process. At the early stage of injury, macrophages can be activated by cytokines to adopt a pro-inflammatory M1 phenotype, while in the middle and later stages of injury, they can be activated to adopt an anti-inflammatory M2 phenotype. M1 macrophages have strong phagocytic and killing abilities, allowing them to phagocytize, ingest and kill pathogenic microorganisms, and other antigens through receptors on the membrane surface and participate in the innate immune response of the body. M1 macrophages are also important antigen-presenting cells that can ingest and process pathogens, present antigens, and stimulate the adaptive immune response. Activated M2 macrophages can secrete a variety of bioactive substances, including cytokines and other complement components, to regulate the immune response and participate in the processes of inflammation, tissue repair and tissue regeneration. When normal tissues are stimulated or damaged by pathogens, growth factors such as TGF-β1 and chemokines can decompose the tissues, promote the translocation of monocytes in the peripheral circulatory system to damaged sites, and stimulate monocytes to further their differentiate into new macrophages. Then, macrophages mediate the immune response, kill pathogens, stimulate angiogenesis, and influence tissue repair (Lingen, 2001). In this study, by double immunofluorescence staining, we observed more M1 macrophages in wound tissues in the PDT treatment groups in the acute inflammatory stage, which indicated that ALA-PDT could promote the activation of macrophages in the early stage of treatment and increase their phagocytic ability. Seven to fourteen days after PDT, the number of M2 macrophages increased significantly, and the M1 macrophages decreased significantly, which was consistent with the proportion of inflammatory factor (IL-1β, TNF-α)/growth factor (TGFβ-1, VEGF) expression. These results suggest that PDT can promote the release of anti-inflammatory factors to weaken the damaging effect of the immune inflammatory response on tissue cells, thus promoting cell proliferation and tissue regeneration.

## Conclusion

In summary, PDT acts in several stages of the healing process and tends to accelerate tissue repair. Our results demonstrate that ALA-PDT can not only kill bacteria but also promote wound healing by regulating inflammatory factors, collagen remodeling and macrophages. This study further clarifies the mechanism of PDT on infectious skin wound healing and provides further experimental evidence for its clinical treatment of skin wounds infected by *P. aeruginosa*. In addition, these results will be of great benefit to expand research on the mechanism by which PDT affects wound healing.

## Data Availability Statement

The original contributions presented in the study are included in the article/supplementary material, further inquiries can be directed to the corresponding author/s.

## Ethics Statement

The animal study was reviewed and approved by the Laboratory Animal Welfare and Ethics Committee of Third Military Medical University.

## Author Contributions

TY, YT, WZ, WY, and XL conceived and designed the study. TY, JL, LC, and HL performed acquisition and analysis of data. JL and GY performed the statistical analysis. YT wrote the first draft of the manuscript. XL provided the administrative, technical, or material support. All authors contributed to the article and approved the submitted version.

## Conflict of Interest

The authors declare that the research was conducted in the absence of any commercial or financial relationships that could be construed as a potential conflict of interest.
